# Region-aggregated attention CNN for disease detection in fruit images

**DOI:** 10.1371/journal.pone.0258880

**Published:** 2021-10-25

**Authors:** Chang Hee Han, Eal Kim, Tan Nhu Nhat Doan, Dongil Han, Seong Joon Yoo, Jin Tae Kwak

**Affiliations:** 1 Department of Computer Science and Engineering, Sejong University, Seoul, Korea; 2 School of Electrical Engineering, Korea University, Seoul, Korea; University of Engineering & Technology, Taxila, PAKISTAN

## Abstract

**Background:**

Diseases and pests have a profound effect on a yearly harvest and productivity in agriculture. A precise and accurate detection of the diseases and pests could facilitate timely treatment and management of the diseases and pests and lessen the resultant loss in economy and health. Herein, we propose an improved design of the disease detection system for plant images.

**Methods:**

Built upon the two-stage framework of object detection neural networks such as Mask R-CNN, the proposed network involves three types of extensions, including the addition of additional level of feature pyramids to improve the exploration and proposal of candidate regions, the aggregation of feature maps from all levels of feature pyramids per candidate region to fully exploit the information from feature pyramids, and the introduction of a squeeze-and-excitation block to the construction of feature pyramids and the aggregated feature maps to improve the representation of feature maps.

**Results:**

The proposed network was evaluated using 74 images of infected apple fruits. In 3-fold cross-validation, the proposed network achieved averaged precision (AP) of 72.26, AP at 0.5 threshold of 88.51 and AP at 0.75 threshold of 82.30. In the comparative experiments, the proposed network outperformed the other competing networks. The utility of the three extensions was also demonstrated in comparison to Mask R-CNN.

**Conclusions:**

The experimental results suggest that the proposed network could identify and localize the symptom of the disease with high accuracy, leading to an early diagnosis and treatment of the disease, and thus holding the potential for improving crop yield and quality.

## Introduction

In agriculture, precise and timely detection of plant diseases and pests is of great importance. Plant diseases and pests could destroy crops, and thus reduce both the quality and quantity of agricultural products, leading to a significant loss in economy and health of our society. Due to the recent climate change, the frequency and variety of plant diseases and pests tend to increase. Early detection of such diseases and pests could aid in developing timely treatment and controlling spread of infection. Visual inspection of plants by an experienced expert has been the most definite means of disease diagnosis, which is time-consuming and expensive. Alternatively, with the widespread of a digital camera, computerized tools that process the digitized images of the plants and perform an automated diagnosis of plant diseases and pests have been developed to improve the accuracy and efficiency of the diagnosis of plant diseases and pests.

**Fig 1:**
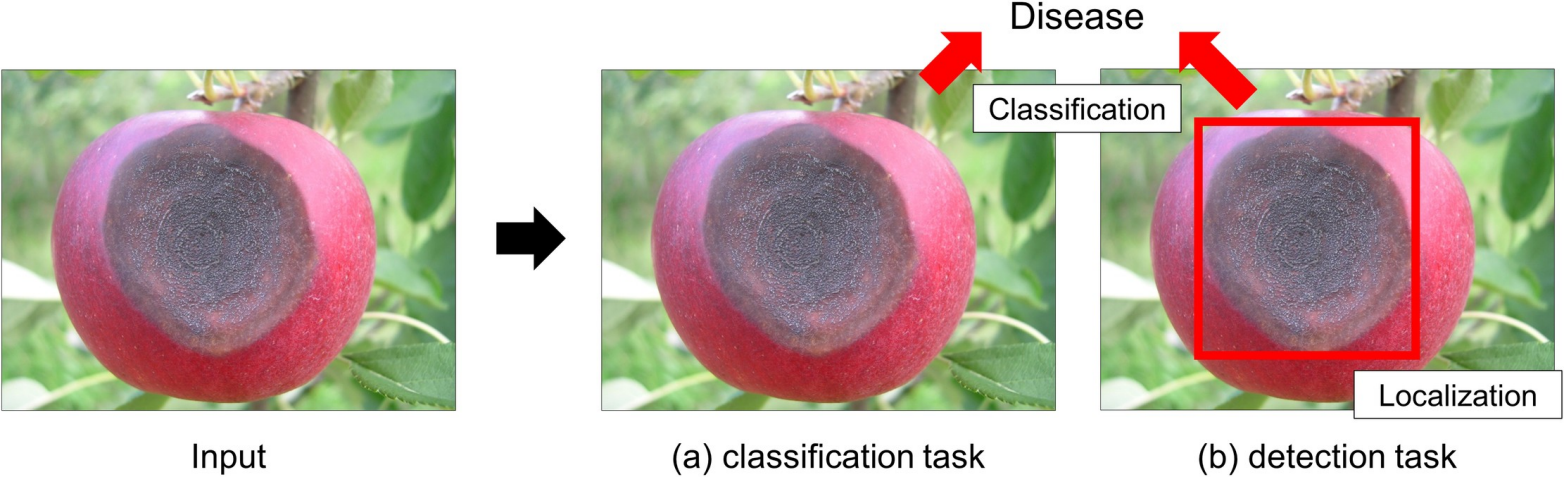
Difference between object classification and detection tasks. Given an input image, (a) an object classification task is to determine whether an image contains the region of the disease of interest and (b) an object detection task is to identify and localize the region of the disease within the image.

Computerized tools are, in general, equipped with several image processing, computer vision and machine learning techniques to improve the quality of plant images, to extract image features and to detect the disease of interest [1]. Machine learning methods include SVM (Support Vector Machine) [2], random forest [3] and ANN (Artificial Neural Network) [4–6]. To characterize and quantify the infected areas, hand-crafted features are often utilized. Hand-crafted features include intensity-based features [6,7], texture features such as GLCM [4,5], Entropy features [6], and shape features [7]. Although a wide variety of hand-crafted features are available, it is impractical to assess all kinds of features and unclear what and how to choose appropriate features. Recently, deep learning has been introduced and outperformed the existing approaches in many applications. A convolutional neural network (CNN) has, in particular, demonstrated a remarkable performance in image recognition and detection tasks [8]. A CNN is capable of extracting image features via a series of convolution and pooling operations without human intervention and classifying an input object into a pertinent class. Several deep learning approaches, based upon a CNN, have been developed for the detection of diseases and pests in agriculture [9]. These are superior to the conventional machine learning approaches [10,11]. However, most of the previous CNN methods only performs a classification task (Fig 1), i.e., given an input image of a plant, it provides a single class label, indicating whether a certain type of disease exists or what type of disease it is. An input image of a plant may contain multiple infected areas of one or more types of diseases. For such a case, it is desirable to identify the exact location of the infected areas, providing much detailed information of the disease and facilitating further analysis of the disease by both human experts and computerized tools.

In this paper, we propose a plant disease detection network that performs both classification and localization tasks (Fig 1). Following a two-stage detector framework of Mask R-CNN [12,13], it proposes candidate regions using bounding boxes at the first stage and identifies the bounding boxes that encompass the infected areas at the second stage. The first stage consists of a feature pyramid network (FPN) [14] that extracts image features at multiple scales from an input image and a region proposal block that identifies bounding boxes that may contain the disease. The second stage contains two branches that conduct a classification of the candidate bounding boxes and a segmentation of the infected areas. The proposed network extends the original two-stage detector framework of Mask R-CNN as follows: (1) feature pyramids are constructed from five levels, not four levels, to better explore the candidate regions for the disease, (2) a bounding box that is identified at one level of FPN is shared with all other levels, and then features are generated from the entire level of feature pyramids and aggregated together to improve feature representations, (3) a squeeze-and-excitation (SE) block [15] is introduced to recalibrate and improve the representation of the aggregated features. The proposed network is evaluated using 74 images of apple fruits.

The experimental results suggest that the proposed network is capable of identifying and localizing the infected areas with high accuracy. The efficacy of the proposed three extensions is also demonstrated in comparative experiments.

The rest of this paper is organized as follows. Related work section reviews the relevant works of deep learning approaches in agriculture. Materials and methods section describes the proposed method in details. Experimental design section presents the experimental settings. Results section demonstrates the experimental results. Discussion section discusses our findings. Conclusion section draws a conclusion.

### Related work

In this section, we provide an overview of deep learning approaches in agriculture as well as a detailed description of a region-based detection network and an attention mechanism that are key aspects of the proposed.

### Application of deep learning in agriculture

Deep learning has been widely applied to numerous fields in agriculture [10], including plant recognition [16] and crop yield estimation [17]. An approach of a CNN has been predominantly adopted for classification tasks. For example, a CNN was successfully adopted for the classification of fine-grained leaf [7] and the classification of 44 different plant species [16]; the classification of crop types was conducted from remote sensing images [18]; the detection of plant diseases and pests was also performed for various types of plants [11]. Moreover, an end-to-end fully convolutional network (FCN) has been applied to various segmentation tasks; for instance, weeds and crop segmentation [19] and leaf segmentation and counting [20]. A CNN has been also introduced to object detection tasks, which perform both classification and localization of an object of interest. A region-based detection network was utilized for fruit counting from orchard imaging data [16] and recognizing tomato plant diseases and pests [21]. In [21], the three existing region-based detection networks, including Single Shot Detector (SSD) [22], R-FCN [23] and Faster R-CNN [24], were adopted and compared to each other. To the best of our knowledge, it was the only attempt to utilize a region-based detection network for the detection of plant diseases and pests.

### Region-based object detection

For object detection, a region- or a region of interest (ROI)-based detection network has been recently proposed and shown to be superior to the previous methods [12]. A region or ROI is defined as a rectangular window of any size within an image. In Fig 1B, the red box illustrates an example of a region or a ROI. R-CNN [12] established the concept of a region-based detection network that feeds region proposal bounding boxes generated by a selective search into a CNN to extract features and to predict pertinent class labels. Since it generates an excessive number of region proposal bounding boxes and examines each box at a time, the detection procedure is extremely slow. Meanwhile, Fast R-CNN [25] was proposed to examine the input image only once and to utilize a region of interest (ROI) pooling layer for an efficient classification and box regression. However, Fast R-CNN still relies on a selective search for region proposals, limiting the speed. To further improve Fast R-CNN, Faster R-CNN [24] was proposed and achieved a (near) real-time object detection. It utilizes a region proposal network (RPN), instead of a selective search, to extract candidate regions. By adopting a set of predefined bounding boxes, called anchor boxes, it efficiently generates boxes of multiple scales and aspect ratios. Furthermore, Mask R-CNN [13], which is the state-of-the-art object detection method, was proposed to perform both instance segmentation (mask branch) as well as object detection (classification branch). It only adds a mask branch, composed of a simplified FCN [26], to Faster R-CNN, yet it achieves a substantial performance gain in object.

### Attention mechanisms

Following the invention of Alexnet [27], composed of a series of multiple convolutional layers, pooling layers and fully-connected layers and a single softmax layer, several improvements have been made to a CNN. Such advances have mainly focused on the design of a network; for example, stacked convolution layers and parallel convolutional layers [28], residual connections [29] and dense connections [30]. Meanwhile, there is another line of research, so-called attention mechanism [31] to recalibrate the given features. Attention mechanism allows a network to utilize the global information of features, to focus on the most informative features, and to suppress less informative features, i.e., improving the efficiency and effectiveness of feature representation of a network. It has already proved its capability in many tasks such as image captioning [32] and image classification [33]. In CNN, attention mechanism can be implemented in two steps–squeeze and excitation (SE) [15]. In a squeeze step, it summarizes the global information of the features and embed them into a set of descriptors. In an excitation step, it recalibrates the features using the descriptors from the squeeze step.

## Materials and methods

Following the framework of Mask R-CNN, the proposed network performs object detection in two stages. The first stage involves an RPN that extracts five-level feature pyramids and proposes candidate regions (for plant disease). The second stage has the network head, equipped with SE and ROI aggregation, that extracts feature maps per candidate box and performs the classification and segmentation of the proposed candidate boxes.

### 1^st^ STAGE: Squeeze-Excitation region proposal network

**Fig 2:**
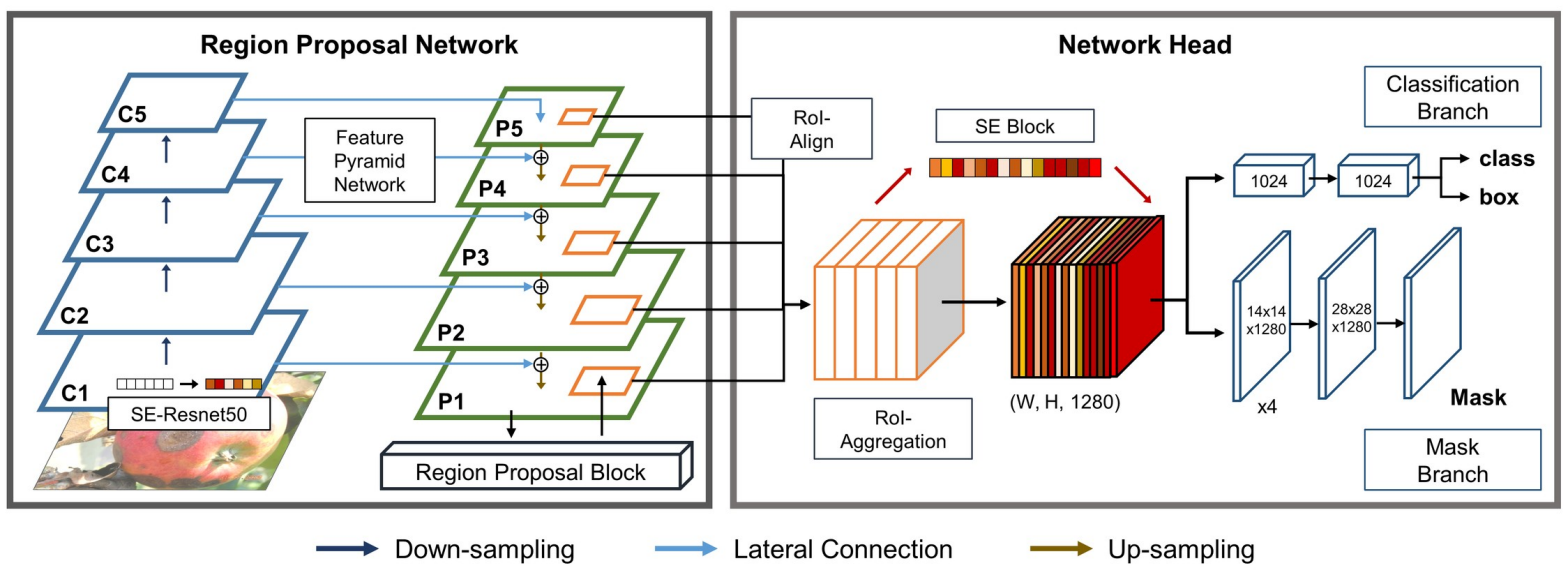
Architecture of the proposed network for the disease detection. It contains two stages. The first stage constructs feature pyramids and propose candidate regions of the disease. The second stage extracts and aggregates feature maps per candidate region and performs the classification and segmentation of the candidate region.

The proposed RPN consists of an FPN and a region proposal block (Fig 2). An FPN extracts features at multiple scales (or levels), involving two pathways–a bottom-up pathway and a top-down pathway. The bottom-up pathway adopts Resnet50 due to its superior performance in the image feature extraction [30]. Resnet50 contains five processing blocks and generates high-level features while successively reducing the spatial size of the input. The first block contains a 7x7 convolutional with a stride of 2. The rest of the blocks are residual blocks where a residual layout of 1x1 convolution, 3x3 convolution, and 1x1 convolution with an identity shortcut connection is utilized. The second, third, fourth, and fifth blocks repeat the residual block 3, 4, 6, and 3 times, respectively. In the second block, a 3x3 max pooling layer with a stride of 2 is added prior to the residual block. The number of output channels of the five blocks are 64, 256, 512, 1024, and 2048 channels, respectively. We denote the output of the five residual blocks as {*C*_1_, *C*_2_, *C*_3_, *C*_4_, *C*_5_}. The top-down pathway obtains the input from the highest level of the bottom-up pathway and upsamples its spatial size by a factor of 2. Then, the output is merged with the output of the lower level of the bottom-up pathway via a later connection. In a later connection, the output of the top-down pathway undergoes a 1x1 convolution and is added to the output of the corresponding level of bottom-up pathway. Repeating the procedure until it reaches the lowest level of the bottom-up pathway, we obtain five feature maps {*P*_1_, *P*_2_, *P*_3_, *P*_4_, *P*_5_}, called feature pyramids.

The original implementation of FPN or RPN utilizes feature pyramids from four levels, i.e., {*C*_2_, *C*_3_, *C*_4_, *C*_5_}, due to memory efficiency, but we utilize the additional level of feature pyramids *P*_1_ for a performance gain. Moreover, we introduce a squeeze-and-excitation (SE) block to RPN. A SE block is known to be useful in recalibrating feature maps, i.e., improving the efficiency and effectiveness of the feature maps at minimal computational cost. A SE block is added to each residual layer of Resnet50.

Given feature pyramids, a region proposal block identifies candidate bounding boxes. It slides an *n* x *n* window over feature pyramids (*n* = 3), extracts a lower-dimensional feature vector per spatial location and conducts a box classification and a box regression (determining the coordinates of a box) in parallel using fully-connected layers. For a box proposal, a single-scale anchor is utilized per pyramid level. The size of the anchors is {32^2^, 32^2^, 64^2^, 128^2^, 256^2^} pixels for {*P*_1_, *P*_2_, *P*_3_, *P*_4_, *P*_5_}, respectively.

### 2^nd^ STAGE: Squeeze-Excitation ROI-Aggregation network head

The second stage includes a ROI-Align-Aggregation (ROIAA) block, a classification branch and a mask branch. Provided with the candidate bounding boxes from the first stage, a ROIAA block extracts feature maps of size *k* x *k* per candidate region. To avoid the issue of quantization, bilinear interpolation is utilized to compute exact feature values per sampled location, and the values are aggregated using max operation to result in *k* x *k* feature maps. As extracting feature maps, we share the candidate bounding boxes with all other levels of feature pyramids, extract feature maps (a spatial size of *k* x *k*) from each level, and aggregate the feature maps via concatenation. Following the concatenation, a SE block is added to recalibrate the aggregated feature maps, i.e., incorporating attention mechanism in the network.

The recalibrated, aggregated feature maps are separately fed into a classification branch and a mask branch. The size of the input feature maps is set to 7x7 (*k* = 7) and 14x14 (*k* = 14) for a classification branch and a mask branch, respectively. A classification branch consists of two convolutions with a 7x7 kernel and a 1x1 kernel, performing a classification of each bounding box and bounding box regression. A mask branch is composed of 4 convolutions with a 3x3 kernel followed by upsampling with a stride 2 and a 1x1 convolution. A mask branch performs a pixel-to-pixel prediction of each bounding box.

### Squeeze-Excitation block

Following [15], we integrate attention mechanism into a SE block. A SE block performs two operations, namely ‘squeeze’ and ‘excitation’. Given a feature map *x*∈ℝ^*H*×*W*×*C*^, the ‘squeeze’ operation conducts global average pooling to summarize the global spatial information of each channel *c* as follows:

$$z_{c}=\frac{1}{H\times W}\sum_{i=1}^{H}\sum_{j=1}^{W}x_{c}(i,j)$$ (1)

where *x*_*c*_ is a *c-*th channel and *H*, *W* and *C* denote a height, width and number of channels, respectively. This squeeze operation provides channel-wise statistics. In the ‘excitation’ operation, a channel descriptor *z*∈ℝ^*C*^ goes through a series of operations to output a transformed channel descriptor *z*′ as follows:

$$z^{\prime }=\rho_{2}\left(\varphi (\rho_{1}(\sigma (z)))\right)$$ (2)

where *σ*, *ρ*, and *φ* denote a sigmoid function, a fully-connected layer (FC), and a rectified linear unit (ReLU), respectively. *ρ*_1_ reduces the dimensionality and *ρ*_2_ increases the dimensionality, i.e., a bottleneck design. Then, we perform channel-wise multiplication between the transformed descriptor *z*′ and the feature map *x*, generating the final output $\tilde{x}=x∙z^{\prime }$.

## Experimental design

### Dataset

74 images of apple fruits were employed to evaluate the performance of the proposed network. Each image contains one or more apples. At least one of them is infected with a disease called *Anthracnose*. The symptom of the disease generally appears rounded with varying sizes and shows visible variations of patterns within the disease. An experienced expert annotated the region of such rounded symptoms on apple fruits with bounding boxes, serving as ground-truth labels for this study. The total number of the bounding boxes is 182, ranging from 1 to 9 per image. The size of apple images ranges from 500x700 to 3000x2000 and the size of disease symptoms ranges from 50x50 to 2000x1400.

### Comparative experiments

To assess the effectiveness of the proposed network, we compare the proposed network to several recently developed object detection methods: 1) Mask R-CNN, 2) single shot multibox detector (SSD) [22], 3) Retinanet [34], and 4) you only look once version 3 (YOLOv3) [35]. Mask R-CNN is a two-stage detector that is composed of RPN and a network head. SDD [22] is a single shot detector for multiple categories. It employs VGG16 [36] to extract feature maps at multiple scales and adopts a set of default bounding boxes and convolutional layers to predict box offsets and category scores. The default bounding boxes are fixed in advanced and have various aspect ratios and scales. Retinanet [34] is a one-stage detector that addresses the extreme foreground-background class imbalance in an object objection task. It utilizes FPN to extract multi-scale feature pyramids and two sub-networks to conduct object classification and box regression, respectively. It also introduces focal loss to force the network focus on hard, misclassified objects during training, leading to an improved accuracy for object detection. YOLOv3 [35] belongs to a family of YOLO [37], which is a one-stage detector that facilitates a real-time object detection of full images. YOLOv3 splits an input image into a grid of cells. For each cell, it predicts bounding boxes, objectiveness, and object class. Darknet-53 is employed a feature extractor and used to extract feature maps at three different scales for the bounding box prediction at those three scales.

## Ablation experiments

The proposed network extends the framework of Mask R-CNN. The extension includes (1) FPN_P_1_: addition of an additional level of feature pyramids, (2) SE_Block: addition of a SE block to FPN (SE_Block_1_) and to aggregated feature maps (SE_Block_2_) and (3) ROI_Agg: aggregation of features from the entire level of feature pyramids. To assess the performance of the proposed network, we compare the proposed network with Mask R-CNN. Using the proposed network, 3-fold cross-validation is performed on the apple dataset where the entire dataset is split into three disjoint subsets, one subset is used as the testing dataset and the other two subsets are used as the training dataset, and the procedure is repeated three times with difference choice of the testing dataset. The identical experiment is repeated for Mask R-CNN.

To further examine the proposed network, the utility of the extended components is also evaluated. We conduct 3-fold cross-validation as adding the extended component to Mask R-CNN as follows: (1) Mask R-CNN + FPN_P_1_, (2) Mask R-CNN + ROI_Agg, (3) Mask R-CNN + FPN_P_1_ + SE_Block_1_, (4) Mask R-CNN + FPN_P_1_ + ROI_Agg, (5) Mask R-CNN + FPN_P_1_ + ROI_Agg + SE_Block_1_, (6) Mask R-CNN + FPN_P_1_ + ROI_Agg + SE_Block_2_.

### Training and inference

We simultaneously train both RPN and the network head. Both networks are optimized via a stochastic gradient descent (SGD) with a batch-size of 2, a learning rate of 0.001, a weight decay of 0.0001, and a momentum of 0.9. Due to memory usage, the size of an input image to a network is resized to 512x512 as maintaining the aspect ratio of the original image. The scaling factor is determined by the shorter side of an image, and the longer side is cropped to 512 pixels after resizing. While training, the following data augmentation techniques are applied: (1) a random cropping in a range from 50 to 80 percent of the size of an input image, (2) a random color variation including brightness in a range of [0.5, 1.5], hue in a range of [–20, 20], saturation in range of [0.8, 1.2], and contrast in a range of [0.75, 1.25]. Moreover, the pre-trained weights, generated on COCO object detection challenge dataset, are used to initialize all the networks used in our experiments. 2000 and 1000 candidate regions are extracted per image during training and validation of each network, respectively. The top-200 candidate regions are fed to a classification branch and a mask branch during training. Training RPN, an anchor is considered as a positive sample (i.e., disease) if intersection over union (IoU) between an anchor with a ground-truth box is higher than a threshold value 0.7 and as a negative sample if an anchor has IoU lower than 0.3 with all ground-truth boxes. For the comparative experiments, all the competing networks are trained with identical settings and procedures. All the networks were implemented using the open-source library Keras and executed on a PC with a TITAN XP GPU.

### Evaluation metrics

To evaluate the performance of the proposed network, we adopt averaged precision over intersection-over-union thresholds (AP), AP at 0.5 IoU threshold (AP_50_) and AP at 0.75 IoU threshold AP_75_). These are commonly utilized for object detection tasks.

## Results

### Results of disease detection

**Fig 3:**
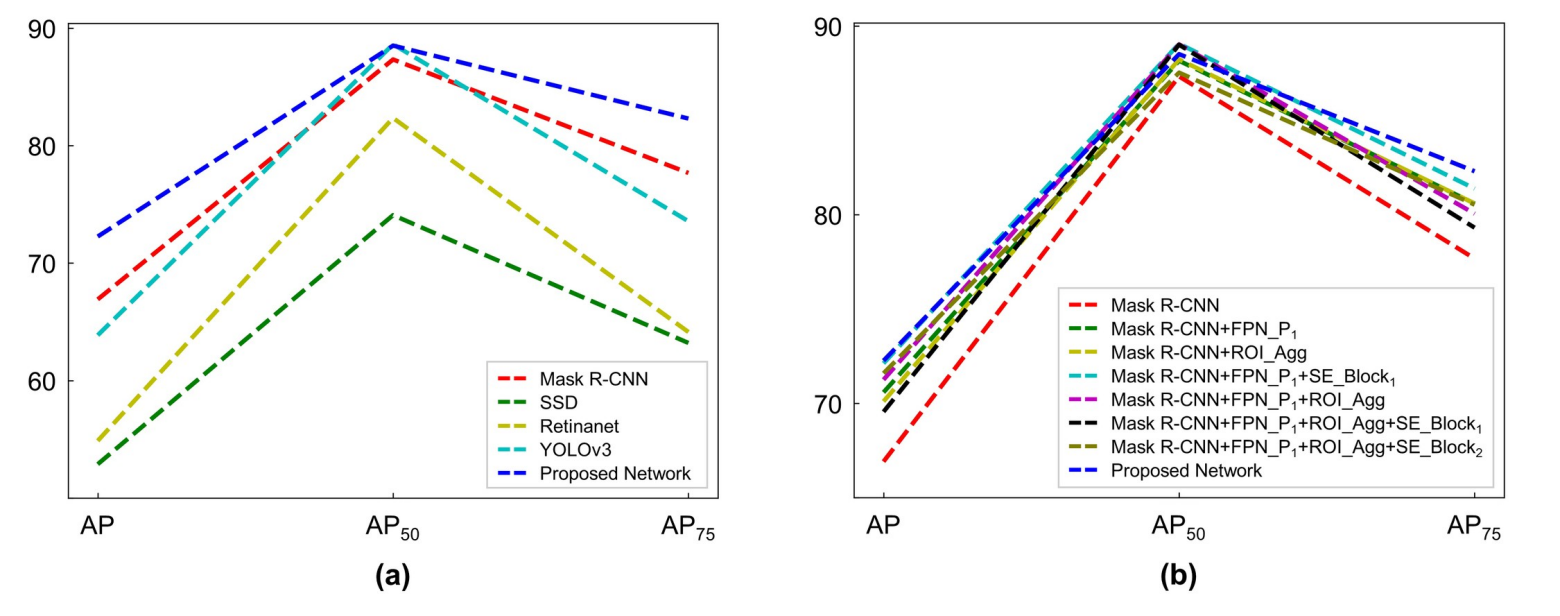
Plots of disease detection results. (a) Results of disease detection by the proposed network and four competing networks. (b) Results of the ablation experiments.

**Fig 4:**
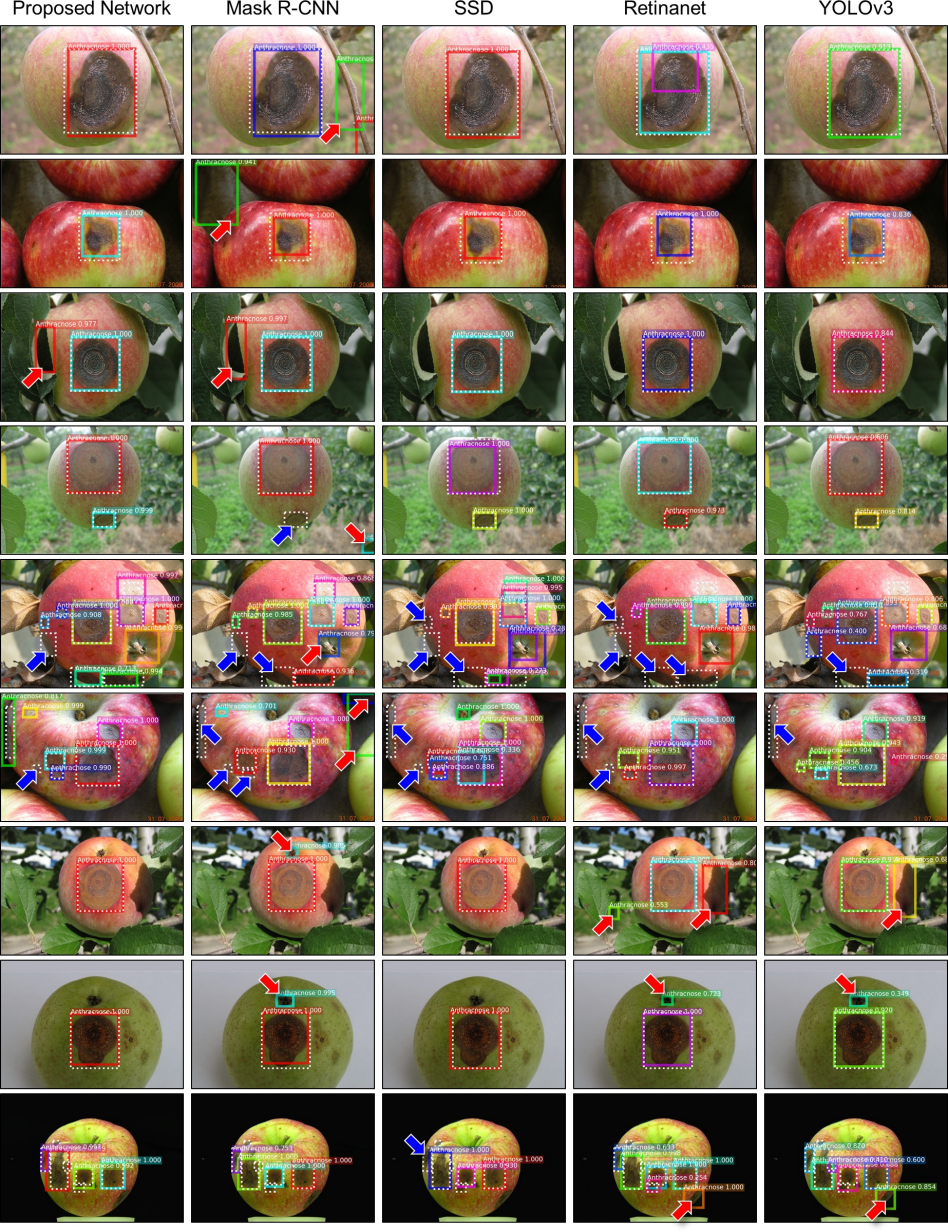
Results of disease detection on apple images. While dotted lines and solid colored lines denote the ground truth and predicted bounding boxes. Red and blue arrows indicate false positive and negative detections, respectively.

Table 1 and Fig 3A show the quantitative results of the proposed network for the detection and localization of the symptom of the disease in apple fruits. In 3-fold cross-validation, the proposed network achieved AP of 72.26, AP_50_ of 88.51 and AP_75_ of 82.30. The qualitative assessment of the results further confirms the robustness and effectiveness of the proposed network. As shown in Fig 4, the proposed network successfully identified the symptom of the disease of varying sizes and shapes under different environments. Although multiple and overlapping symptoms of the disease were present in a single input image, the proposed network was able to classify and localize them with high accuracy.

**Table 1:** Results of disease detection.

Model	AP	AP_50_	AP_75_
Mask R-CNN	66.91	87.33	77.67
SSD	52.88	74.08	63.29
Retinanet	54.87	82.35	64.13
YOLOv3	63.86	88.62	73.56
Proposed Network	72.26	88.51	82.30

### Comparative experiments

In the comparative experiments, the proposed network outperformed four competing models, including Mask R-CNN, SSD, Retinanet, and YOLOv3 for all evaluation metrics except AP_50_ by YOLOv3 (Table 1 and Fig 3A). In comparison to the proposed network, YOLOv3 improved AP_50_ by 0.11 but substantial decreased AP and AP_75_ by ≥8.40. Among the competing models, SSD and Retinanet showed the worst performance. Mask R-CNN was, in general, superior to other competing models, however, the proposed network outperformed Mask R-CNN across all the evaluation metrics, improving AP, AP_50_ and AP_75_ by 5.35, 1.18 and 4.63, respectively. These results indicate that the two-stage detectors (Mask R-CNN and the proposed network) are, by and large, superior to the one-stage detectors with respect to the detection quality.

### Ablation experiments

Table 2 and Fig 3B show the results of ablation experiments where the effectiveness of the extended components of the proposed network was investigated. The addition of an additional level of feature pyramids (FPN_P_1_) to Mask R-CNN (Mask R-CNN+FPN_P_1_) outperformed Mask R-CNN: increase in AP, AP_50_ and AP_75_ by 3.67, 0.83 and 2.92, respectively. The introduction of the feature map aggregation (ROI_Agg) to Mask R-CNN (Mask R-CNN+ROI_Agg) and Mask R-CNN+FPN_P_1_ (Mask R-CNN+FPN_P_1_+ROI_Agg) further improved the performance (Mask R-CNN: AP by 3.09, AP_50_ by 0.90 and AP_75_ by 2.92 and Mask R-CNN+FPN_P_1_: AP by 1.54, AP_50_ by 0.90 and AP_75_ by 0.79). Moreover, the addition of a SE block to FPN (SE_Block_1_) in Mask R-CNN+FPN_P_1_ (Mask R-CNN+FPN_P_1_+SE_Block_1_) resulted in an improvement on AP, AP_50_ and AP_75_ by 1.54, 0.90 and 0.79, respectively. However, the combination of a SE block (SE_Block_1_ or SE_Block_2_) with ROI_Agg was not always able to provide a performance gain. Mask R-CNN+FPN_P_1_+ROI_Agg+SE_Block_1_ was inferior to Mask R-CNN+FPN_P_1_+ROI_Agg. Mask R-CNN+FPN_P_1_+ROI_Agg+SE_Block_2_ only increased AP by 0.34 and AP_75_ by 0.48 in comparison with the network without SE_Block_2_.

**Table 2:** Results of ablation experiments.

Model	AP	AP_50_	AP_75_
Mask R-CNN	66.91	87.33	77.67
Mask R-CNN+FPN_P_1_	70.58	88.16	80.59
Mask R-CNN+ROI_Agg	70.10	88.23	80.59
Mask R-CNN+FPN_P_1_+SE_Block_1_	72.12	89.06	81.38
Mask R-CNN+FPN_P_1_+ROI_Agg	71.25	89.03	80.05
Mask R-CNN+FPN_P_1_+ROI_Agg +SE_Block_1_	69.55	89.01	79.31
Mask R-CNN+FPN_P_1_+ROI_Agg +SE_Block_2_	71.59	87.53	80.53
Proposed Network	72.26	88.51	82.30

### Network analysis

We further assessed the utility of FPN_P_1_, adding the additional level of feature pyramids, i.e., *P*_1_ to FPN, in the proposed network. It has a direct relationship with a region proposal block, proposing candidate regions, and a ROIAA block, extracting feature maps from the candidate regions. The effect of the additional feature maps from *P*_1_ was evaluated above. In order to investigate the effect of FPN_P_1_ on a region proposal block, the fraction of the candidate regions that were generated from *P*_1_ was computed and compared using Mask R-CNN+FPN_P_1_ at inference (proposing 1000 regions). As shown in Table 3, on average, 45% of the candidate regions were obtained from *P*_1_. Moreover, the number of candidate regions with high confidence (the probability of a box classification > 0.7) was measured. The number of candidate regions generated from *P*_1_ comprised 83% of the entire number of candidate regions with high confidence (Table 3).

**Table 3:** Number of candidate regions.

Level	Avg. # of Candidate Regions (%)	Avg. # of Candidate Regions with High Confidence (%)
P_1_	453.8 (45.38%)	8.52 (83.03%)
P_2_	310.64 (31.06%)	1.21 (11.72%)
P_3_	194.70 (19.47%)	0.41 (4.2%)
P_4_	33.86 (3.38%)	0.04 (0.34%)
P_5_	6.98 (0.69%)	0.04 (0.67%)

## Discussion

This study has investigated an approach of advanced deep learning networks to disease detection in plant images. The experimental results demonstrated that the proposed network could identify and localize the disease in a plant image with high accuracy. The detection results of the proposed network were robust to variations in the number, size and shape of the symptom of the disease, suggesting that the network was capable of capturing the underlying characteristics of the symptom of the disease.

To build the proposed network, three types of extensions were made to Mask R-CNN, which is the state-of-the-art objection detection method. Owing to the extensions, the performance of the proposed network was superior to that of Mask R-CNN. Each of the extensions contributed to the performance gain obtained by the proposed network, but their effect was disproportionate. The result of adding FPN_P_1_, SE_Block_1_, and ROI_Agg was incremental for all the evaluation metrics, whereas the effect of SE_Block_1_ and SE_Block_2_ was indistinct, observing no or minimal increase in AP and AP_50_ and a slight decrease in AP_75_. Nevertheless, the proposed network, combining all the extensions, obtained the best overall performance.

Attention mechanism has been successfully applied to many applications, including object detection in synthetic aperture radar (SAR) images [38], image classification in hyperspectral images [39], and image segmentation in magnetic resonance imaging (MRI) [40]. Given feature maps, most of attention mechanisms squeeze the global channel information, by using global average pooling, and excite them channel-wise, and thus it is also called as a channel SE block. Moreover, alternative SE blocks have been proposed. For example, a spatial SE block [39,41] squeezes along the channel and excites spatially. A concurrent channel and spatial SE block [39,41] separately conducts a channel-wise and spatial-wise SE and combines them together via max-out, addition, multiplication, or concatenation. Some others also performed a channel-wise SE and a spatial-wise SE in a row [32]. In this study, we employed the original implementation of the SE block, i.e., a channel SE block. The integration of other variants of SE blocks may provide an additional performance gain. However, it is unclear what variants or combinations would result in the best performance. Optimizing the SE block is not the scope of this study. Hence, we leave this for the future study.

Several false detections were made by the proposed network. We observed that there was a general trend of the false detections. First, the appearance of the symptom of the disease was similar to that of non-disease areas. Many of the symptom of the disease is darker than the surrounding areas, and thus the dark background or objects such as leaves could have similar characteristics with the disease. Second, non-disease areas with irregular patterns such as the edge and the stalk of an apple were often classified as disease. Third, smaller symptoms of the disease were harder to detect than the symptoms of intermediate or large size. Last, ambiguous annotations caused confusion. Several images have overlapping regions of the symptom of the disease. The exact boundary between the overlapping regions is unclear, and thus the proposed network could make over- or under-detections, leading to false detections.

In a head-to-head comparison of the detection results between the proposed network and other competing models, the superiority of the proposed network was further highlighted. Similar to the proposed network, other competing models often missed small symptoms of the disease and identified non-disease areas with irregular patterns or dark background as disease. In particular, dark background and/or leaves at the corner or near the border of an input image were often mis-classified whereas the proposed network correctly classified them. The improvement in disease detection may be ascribable to both stages of the proposed network. The first stage, equipped with a SE block and an additional level of feature pyramids, facilitates an improved exploration of candidate regions, and thus reduces false negative detections. The second stage, combining a SE block and a ROI aggregation, allows the network to obtain and recalibrate multiscale feature maps, leading an improved classification of candidate regions, i.e., reducing both false negative and positive detections.

The proposed network was only evaluated using a small dataset. Although the dataset includes images taken under different environments, the efficacy of the proposed network on the independent dataset has not been assessed. Moreover, the proposed network has been only evaluated on apple images. As other fruit images of similar quality with the apple images in this work are provided, the proposed network should be able to detect the areas of disease in other fruit images. Nevertheless, a large-scale study should be followed to further ensure the reliability and validity of the proposed network for the detection of disease in fruit images.

## Conclusion

In this paper, we proposed a disease detection network that performs both classification and localization of the symptom of the disease in a plant image. With the advanced design of the network, we were able to achieve a precise and robust detection of the disease, outperforming the current state-of-the-art objection detection method. The effectiveness and utility of the advances in the network design were confirmed by the comparative experiments. The ability to accurately identify and localize the disease in a plant image could facilitate early diagnosis and treatment of the disease, holding the potential for improving crop yield and quality. The framework of the proposed network is generic, and thus could be applied to other types of images and object detection tasks.
